# VA’s Life-Sustaining Treatment Decisions Initiative: First 20 Months of National Implementation

**DOI:** 10.1093/geroni/igaa057.2709

**Published:** 2020-12-16

**Authors:** Mary Ersek, Winifred Scott, Joan Carpenter, Jennifer Kononowech, Ciarian Phibbs, Jennifer Cohen, Mary Beth Foglia, Cari Levy

**Affiliations:** 1 Department of Veterans Affairs, Philadelphia, Pennsylvania, United States; 2 VA Palo Alto Healthcare System, Palo Alto, California, United States; 3 Department of Veterans Affairs, Berlin, Maryland, United States; 4 VA Ann Arbor Healthcare System, Ann Arbor, Michigan, United States; 5 VA Palo Alto Healthcare System, Menlo Park, California, United States; 6 VA Puget Sound Health Care System, Seattle, Washington, United States; 7 VA - National Center for Ethics in Health Care, Seattle, Washington, United States; 8 VA Eastern Colorado Health Care System, Aurora, Colorado, United States

## Abstract

This retrospective observational study describes the first 20 months of implementing the Life-Sustaining Treatment Decisions Initiative. We examined patient and facility characteristics associated with life sustaining treatment (LST) orders template completion, including the association between template completion and the Care Assessment Need (CAN) score, which quantifies Veterans’ risk of hospitalization and mortality. As of February 29, 2020, over 274,200 Veterans received at least one goal of care conversation and LST preferences documented on a template. Eighty-two percent of deceased Veterans with the highest risk of hospitalization or mortality had an LST note and order documented prior to their death. Factors that predicted a greater likelihood of LST template completion included higher CAN score, older age, nursing home stay, and being white non-Hispanic. Findings suggest that clinicians are engaging older, sicker veterans in goals of care conversations. Research is needed to understand potential disparities in LST template completion.

